# Rapidly growing nodule on the finger of a pregnant woman

**DOI:** 10.4103/0256-4947.72283

**Published:** 2010

**Authors:** Yousef Binamer

**Affiliations:** From the Department of Medicine, Division of Dermatology, McGill University, Montreal, Québec, Canada

A 27-year-old woman, 28-weeks pregnant, presented with a rapidly growing nodule on her finger with spontaneous bleeding over the previous 4 weeks. Physical exam revealed a pedunculated vascular tumor on the left index finger (**Figure 1**).

The most likely diagnosis is:

**Figure 1:**
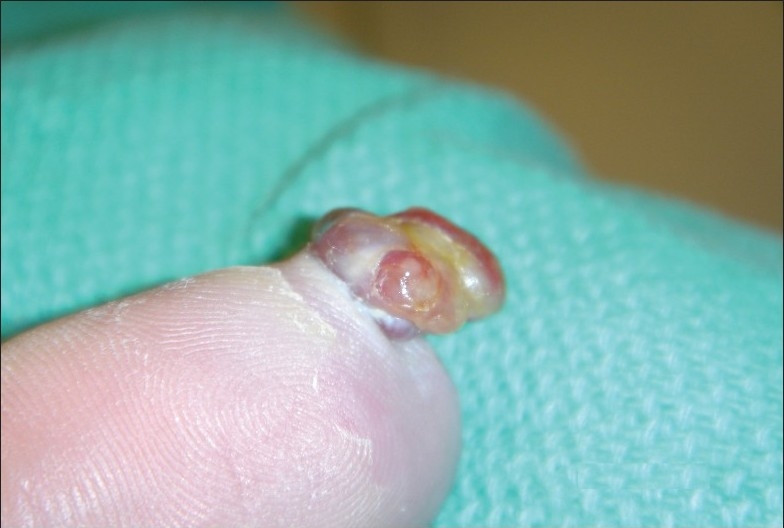
The nodule.

- Malignant melanoma
- Pyogenic granuloma
- Basal cell carcinoma
- Orf

## Diagnosis: Pyogenic granuloma

Pyogenic granuloma (PG) is a common vascular tumor. PG is a misnomer, as it is neither infec-tious nor granulomatous in origin. It is con-sidered to be a process reactive to an unknown factor, which could be traumatic or hormonal. It is commonly seen in children and adolescents, as well as in pregnant women. Multiple drugs have been reported to cause PG, including oral and topical retinoids, ritonavir, epi-dermal growth factor receptor blockers and 5-fluoro-uracil.^1^–^3^ Other uncommon variants are disseminated, subcutaneous and intravenous forms. It presents as rap-idly growing beefy red papules that form a pedunculat-ed nodule with a collarette of scales that often become ulcerated. It varies in size from a few millimeters to a few centimeters. The most common areas involved are the head and neck, followed by the extremities. Since bleeding is a very common feature, the patient gener-ally covers it with an adhesive bandage. Therefore, some authors have called this “the band-aid sign.”^4^ However, the occurrence of such rapidly growing lesion in adults should alert the health care provider to other possibili-ties, including malignant melanoma.

PG should be differentiated from other mimickers, such as angiolymphoid hyperplasia with eosinophilia, hemangioma, malignant melanoma and basal cell car-cinoma. Malignant melanoma usually has a pigmented component, as well as less bleeding tendency. Other vas-cular tumors are less likely to be seen during pregnancy.

PG does not involute spontaneously. Different treatment modalities have been described, including surgical excision, curettage, electrodessication, cautery via silver nitrate, pulse dye laser, cryotherapy, and topi-cal imiquimod 5% cream.^5^ However, surgical excision with cauterization of the base is the preferred method if the health care provider is in doubt about the diagnosis.

FOR THE ANSWER, VISIT: http://www.saudiannals.net
